# Tracking implementation strategies: a description of a practical approach and early findings

**DOI:** 10.1186/s12961-017-0175-y

**Published:** 2017-02-23

**Authors:** Alicia C. Bunger, Byron J. Powell, Hillary A. Robertson, Hannah MacDowell, Sarah A. Birken, Christopher Shea

**Affiliations:** 1 College of Social Work, The Ohio State University, 1947 College Road, Columbus, OH 43210 United States of America; 2 Department of Health Policy and Management, Gillings School of Global Public Health, University of North Carolina at Chapel Hill, 1105C McGavran-Greenberg Hall, Campus Box 7411, Chapel Hill, NC 27599 United States of America; 3 Georgetown University Center for Child and Human Development, Department of Pediatrics, Georgetown University, 3300 Whitehaven Street, NW, Suite 3300, Washington, DC 20007 United States of America

**Keywords:** Implementation strategies, Methods, Measurement, Reporting

## Abstract

**Background:**

Published descriptions of implementation strategies often lack precision and consistency, limiting replicability and slowing accumulation of knowledge. Recent publication guidelines for implementation strategies call for improved description of the activities, dose, rationale and expected outcome(s) of strategies. However, capturing implementation strategies with this level of detail can be challenging, as responsibility for implementation is often diffuse and strategies may be flexibly applied as barriers and challenges emerge. We describe and demonstrate the development and application of a practical approach to identifying implementation strategies used in research and practice that could be used to guide their description and specification.

**Methods:**

An approach to tracking implementation strategies using activity logs completed by project personnel was developed to facilitate identification of discrete strategies. This approach was piloted in the context of a multi-component project to improve children’s access to behavioural health services in a county-based child welfare agency. Key project personnel completed monthly activity logs that gathered data on strategies used over 17 months. Logs collected information about implementation activities, intent, duration and individuals involved. Using a consensus approach, two sets of coders categorised each activity based upon Powell et al.’s (Med Care Res Rev 69:123–57, 2012) taxonomy of implementation strategies.

**Results:**

Participants reported on 473 activities, which represent 45 unique strategies. Initial implementation was characterised by planning strategies followed by educational strategies. After project launch, quality management strategies predominated, suggesting a progression of implementation over time. Together, these strategies accounted for 1594 person-hours, many of which were reported by the leadership team that was responsible for project design, implementation and oversight.

**Conclusions:**

This approach allows for identifying discrete implementation strategies used over time, estimating dose, describing temporal ordering of implementation strategies, and pinpointing the major implementation actors. This detail could facilitate clear reporting of a full range of implementation strategies, including those that may be less observable. This approach could lead to a more nuanced understanding of what it takes to implement different innovations, the types of strategies that are most useful during specific phases of implementation, and how implementation strategies need to be adaptively applied throughout the course of a given initiative.

**Electronic supplementary material:**

The online version of this article (doi:10.1186/s12961-017-0175-y) contains supplementary material, which is available to authorized users.

## Background

Persistent gaps between what we know and what we do in behavioural health and social services has led to the prioritisation of implementation research [1–3], which is defined as “*the scientific study of methods to promote the systematic uptake of research findings and other evidence-based practices into routine practice*” [4]. One of the central concerns of implementation research is developing a better understanding of implementation strategies, which we define as methods or techniques used to enhance the adoption, implementation and sustainment of a program or practice [5]. Over 70 discrete implementation strategies have been identified (e.g. audit and feedback, educational workshops, facilitation, supervision) [6, 7], and evidence for their effectiveness continues to accumulate [8–10]. There is increasing consensus that improving implementation outcomes (e.g. adoption, fidelity, penetration, sustainability) [11], particularly for complex interventions, will require that multiple discrete strategies be selected and tailored to address the multilevel determinants (i.e. barriers and facilitators) of implementation [12–16]. To understand what it takes to effectively implement programs and practices in behavioural health and social service settings, a number of experimental (e.g. [17–24]), quasi-experimental (e.g. [25, 26]) and observational (e.g. [27–29]) studies have been conducted.

Despite the growth in empirical studies, published reports of implementation often fail to provide detailed descriptions of implementation strategies [10, 27]. The poor quality of reporting in published implementation studies limits replication of specific implementation strategies in both research and practice, and hinders our ability to understand how and why implementation strategies are successful. Failing to understand what investigators and other stakeholders did to implement a given program or practice also constrains our ability to interpret implementation successes or improve upon implementation failures. Furthermore, poor reporting limits our ability to learn across studies in systematic reviews, meta-analyses, and other forms of research synthesis. For example, a recent systematic review of quality improvement collaboratives concluded that “*reporting on specific components of the collaborative was imprecise across articles, rendering it impossible to identify active quality improvement collaborative ingredients linked to improved care*” [28].

One approach to improving the quality of reporting in implementation research has been to develop more standardised language for implementation strategies [6, 7, 29]. For example, Powell et al. [6] developed a compilation of 68 discrete implementation strategies that were categorised into six taxonomic headings, namely (1) planning, (2) educating, (3) financing, (4) restructuring, (5) managing quality, and (6) attending to the policy context. Another approach has been to advance various reporting guidelines that specify elements of interventions, implementation strategies and other aspects of implementation (e.g. outcomes, context) that need to be carefully described to maximise consumers’ ability to benefit from published implementation studies [5, 30–32]. For example, Proctor et al. [5] advanced guidelines that suggest that researchers should name and define implementation strategies in ways that are consistent with the published literature, and carefully specify the following elements: (1) actor (i.e. who enacts the strategy?), (2) action(s) (i.e. what are the specific actions, steps, or processes that need to be enacted, (3) action target (i.e. what constructs are targeted? What is the unit of analysis?), (4) temporality (i.e. when is the strategy used?), (5) dose (i.e. what is the intensity?), (6) implementation outcome (i.e. what implementation outcome(s) are likely to be affected by each strategy?), and (7) justification (i.e. what is the empirical, theoretical, or pragmatic justification for the choice of implementation strategy?). Applied examples of this approach demonstrate its utility in improving the clarity of implementation strategies and maximising the potential that they can be replicated in research and practice [33, 34].

The development of taxonomies and reporting guidelines has certainly played a role in advancing clarity in the field; however, to truly improve our understanding of when, where, why and how implementation strategies are effective in promoting implementation and clinical outcomes, these approaches will need to be complemented by practical approaches for tracking implementation strategies within the context of implementation studies and ‘real world’ implementation efforts. Indeed, practical approaches for identifying and describing implementation strategies have been identified as a priority for the field [35–38]. Tracking implementation strategy use over time can lead to a more nuanced understanding of what it takes to implement different innovations, the types of strategies that are most useful during specific phases of implementation, and how implementation strategies need to be adaptively applied throughout the course of a given initiative.

The purpose of this naturalistic, observational study is to identify and describe the strategies used to implement a multicomponent intervention in a way that facilitates clear reporting and specification. Specifically, this article (1) describes the application of a practical approach using activity logs to track strategies in an ongoing implementation initiative, and (2) summarises the strategies used in the initiative.

## Methods

### Study context and implementation tracking goals

**Fig. 1:**
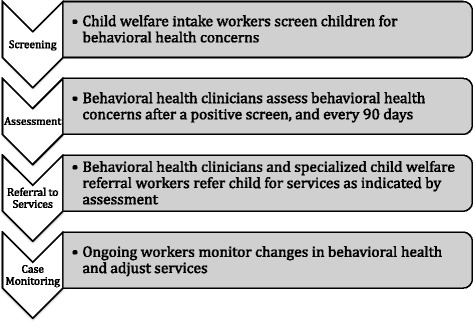
Project description

This study was set in the context of a system demonstration project intended to improve access to behavioural health services for child welfare-involved youth. The project is being conducted in one site, a large United States county-based child welfare agency (more than 300 front-line workers) in a Midwestern state. A core leadership team of high-level implementation actors was assembled from the project start to lead design and implementation. Although the team fluctuated in terms of size and composition (given turnover and the needs of the project), there has been consistent representation by senior administrators from the child welfare agency, an administrator from the co-located behavioural health assessment team, contracted project management staff, and external and internal evaluators. During the first project phase (October 2012 to April 2014), an extensive needs assessment was conducted to inform the design. Beginning in May 2014, the leadership team focused on planning and designing four project components that involve the implementation of new practices and routines for identifying children with behavioural health needs and connecting them to services, including screening, assessment, referrals to community-based services, and on-going case monitoring (Fig. 1; Additional file 1).

Leading up to the project launch in February 2015, the leadership team delegated implementation planning to four additional implementation teams (corresponding to each of the four project components). These implementation teams provided input, redesigned workflow processes and executed the implementation plan elements; thus, these groups identified and used implementation strategies. Although the size and formality varied, each implementation team was comprised of mid-level administrators and supervisors from the child welfare and behavioural health agency units responsible for each particular project component.

### Development of our strategy tracking approach

Our goal was to capture and report how project components were implemented to facilitate their replication elsewhere. Since the leadership and implementation teams’ meetings were the primary venue for identifying and discussing implementation strategies, we originally planned to capture this information at the end of the project by examining meeting minutes and conducting key informant interviews. However, we quickly learned about the substantial time leadership and implementation team members devoted to implementation outside of meetings (drafting educational materials, developing quality assurance procedures for monitoring the new screening and assessments results, and building buy-in among front-line staff). We doubted that our original approach would yield complete or sufficiently detailed information about the full range of discrete strategies used due to the number of efforts that occurred, the fact that strategies were not always discussed within formal meetings, and the chances that all efforts might not be remembered accurately.

As an alternative, we explored existing tools such as the Stages of Implementation Completion observational measure [35, 39] and the Stages of Implementation Analysis [40], which track progress through implementation stages. Yet, their focus on achievement of pre-determined, observable implementation milestones (e.g. clinical training) may not capture the more nuanced strategies occurring ‘behind the scenes’. Thus, we developed a strategy tracking approach to generate more detailed information about the types of implementation strategies used. Given their utility for process evaluation, we developed an activity log for the members of the leadership and implementation teams to record their implementation activities (actions, methods, events, or efforts to promote adoption and implementation of project components). In this log, team members were instructed to list each implementation activity they engaged in over the last month. For each activity, team members recorded the purpose (to identify the type of strategy), estimated length of time (to estimate dosage), and individuals involved (to specify actors) (Additional file 2) [41]. This approach was designed to gather retrospective and prospective data from a variety of project personnel that would fulfil our funders’ request for detailed description of implementation in a way that is flexible, low-cost and practical for our project partners. We used these logs to identify implementation strategies used by the leadership and implementation teams retrospectively during an earlier 9-month implementation planning phase (May 2014 to January 2015), and prospectively during the first 8 months of active implementation (February 2015 to September 2015). Thus, our data span 17 months.

### Participants

Participants included 15 individuals who participated in the leadership or four implementation teams over 17 months. Thus, participants represent multiple respondents from our single site, and included the project director, project managers, internal evaluators, associate directors and supervisors from the county child welfare agency, as well representatives from the behavioural health partner and an external evaluator. Implementation activity logs and consent to participate were requested from key stakeholders each month. The number of participants fluctuated between 13 and 14 per month depending on staff turnover. The response rate was 92.31% for retrospective data collection (May 2014 to January 2015) and 75% on average each month for data collected prospectively February to September 2015.

### Data collection procedures

Data on implementation activities were collected in two stages.

#### Retrospective data collection (implementation planning: May 2014 to January 2015)

Implementation activities that occurred during the months of May 2014 to January 2015 were collected retrospectively in February 2015. Prior to data collection, the evaluation team met with the leadership and four implementation teams during regular meetings to explain the rationale, the proposed methods for our strategy tracking approach and to answer questions. To facilitate recall, participants were encouraged to review their calendars and project-files during these planning months. Participants were given the option of completing an activity log for all 9 months or reviewing their activities month by month over the phone with a member of the research team (these calls lasted 30–60 minutes).

#### Prospective data collection (active implementation: February 2015 to September 2015)

From February 2015 onwards, data on implementation activities were gathered prospectively. Members of the leadership and implementation teams were sent a monthly email requesting that they complete an activity log, which took 15–30 minutes to complete depending on the number of activities to report. Initially, participants had difficulty distinguishing implementation-related activities from other service delivery or administrative tasks (e.g. routine staff meetings or non-project related case consultation). Therefore, we asked participants to take an inclusive approach and include any activities they believed were related to implementation, so that the evaluation team could make a determination. We also added a hypothetical example to the instructions, and shared the list of discrete implementation strategies compiled in the Powell et al. [6] taxonomy. Up to two monthly reminder emails were sent, and in-person reminders were provided during regular meetings. Each month, the research team invested up to 2 hours to request the logs, follow-up with participants and address participants’ questions.

### Coding

Each month, activities collected in individual logs were combined and reviewed. Duplicate activities (e.g. monthly leadership meetings that were reported by multiple participants) were combined into a single entry. If sufficient explanation of the activity was not provided in the log, a research team member followed up with the participant to clarify, although this was rare. First, the team assigned codes for each reported activity based upon the team or type of implementation actors involved (leadership, screening, assessment, referral and case monitoring implementation teams).

Second, two members of the research team (AB and HR) reviewed each month’s activities and coded them using Powell et al.’s [6] taxonomy of implementation strategies as a codebook. We used the taxonomy of Powell et al. [2] because we were most familiar with the strategies and their definitions. For additional detail and examples of how the Powell et al. compilation [6] was used as a codebook, see Additional file 3. It should be noted that that the Powell et al. [6] taxonomy has been updated, although strategy tracking and coding in our project began prior to the publication of the new compilation [7, 42].

During coding, we noticed that some activities included multiple discrete strategies and thus a single activity could have multiple codes (e.g. a meeting among the intake unit supervisors and project managers to detail new workflow processes and strategies for building staff buy-in were coded as ‘planning – tailor strategies to overcome barriers and honour preferences’ and ‘planning – identify and prepare champions’). To enhance the validity of the coding process, a third coder (HM) also reviewed and coded the activity logs independently. Both sets of coded logs were compared; there was a high degree of discordance (e.g. 50% agreement for May 2014) reflecting complexities in the data, and difficulties in applying strategy taxonomies [43]. Therefore, a consensus-based approach to coding was needed. For each month of implementation logs, the three coders met (for up to 2 hours), discussed discrepancies, developed a shared understanding of each strategy, clarified how each strategy definition in the Powell et al. [6] taxonomy applied in this unique project context, and reached consensus. We found that these consensus meetings were needed throughout coding because as we reached consensus on a set of codes for activities used during the earlier stages (e.g. planning strategies), we would observe an influx of different activities as implementation progressed (e.g. quality management) requiring additional conversation and clarification.

### Analysis

Each activity reported was entered into a data file along with the assigned strategy codes and other information (e.g. number of participants, amount of time, project component). We used univariate techniques to explore and describe the strategy types, temporality, dosage and actors using Stata v14.

## Results

Participants listed 473 unique implementation activities on their logs from May 1, 2014, through September 30, 2015. About half of these activities represented one discrete implementation strategy from Powell et al.’s [6] compilation, 33% combined two strategies, and 17% incorporated three or more strategies. Thus, across the 473 activities, participants used implementation strategies 611 times.

### Types of implementation strategies

First, data were used to identify types of implementation strategies (the actions in Proctor et al.’s [5] specification guidelines). Of the 68 strategies included in Powell et al.’s [6] compilation, 43 distinct implementation strategies were identified in the data during the 17-month implementation period (Table 1). Two additional strategies (obtained worker feedback about the implementation plan and plan for the outcome evaluation) emerged during data coding that did not fit within the existing compilation. The first new strategy, obtaining worker feedback involved a variety of formal and informal conversations with front-line workers that solicited their opinions about the implementation plan. This information was often shared with the implementation and leadership teams and used to make adjustments. The second strategy, plan for outcome evaluation, might be specific to the unique context of this project (an outcome study is required by the project funder, but might not be conducted under different circumstances). There were several meetings and phone conversations among project staff, and the internal and external evaluators to identify relevant constructs, measures and data sources, and ensure alignment between plans for internal quality management and the outcome evaluation.

**Table 1:** Frequency of discrete implementation strategies used (n = 611)

Strategy	f	%
*Planning*	184	30.11%
Tailor strategies	86	14.08%
ID and prep champions	43	7.04%
Develop blueprint	36	5.89%
Build buy-in	35	5.73%
Assess readiness, ID barriers	29	4.75%
Recruit, train leadership	14	2.29%
Planning (general)	7	1.15%
Select strategies (general)	4	0.65%
Stage scale up	4	0.65%
Consensus discussions	4	0.65%
Involve executive boards	4	0.65%
Conduct local needs assessment	3	0.49%
Visit other sites	3	0.49%
Academic partnerships	2	0.33%
Gather information (general)	1	0.16%
Develop relationships (general)	1	0.16%
*Education*	105	17.18%
Informal local opinion leaders	43	7.04%
Conduct educational meetings	19	3.11%
Distribute materials	14	2.29%
Develop effective materials	12	1.96%
Conduct ongoing training	12	1.96%
Ongoing consultation	7	1.15%
Inform & influence stakeholders	7	1.15%
Work with education institutions	2	0.33%
Develop glossary	1	0.16%
Educate through peers (general)	1	0.16%
*Finance*	14	2.29%
Fund/contract	13	2.13%
Access new funding	1	0.16%
*Restructure*	44	7.2%
Change records systems	29	4.75%
Change structure/equipment	15	2.45%
Revise roles	2	0.33%
*Quality Management*	264	43.2%
Develop systems	53	8.67%
Use data experts	48	7.86%
Clinical supervision	47	7.69%
Develop tools	42	6.87%
Reminders	29	4.75%
Purposefully re-examine implementation	28	4.58%
Obtain worker feedback^a^	28	4.58%
Plan for outcome evaluation^a^	18	2.95%
Audit & feedback	15	2.45%
Data warehouse	14	2.29%
Organise implementation team meetings	11	1.80%
Centralise technical assistance	10	1.64%
Capture and share local knowledge	9	1.47%
Use advisory boards	5	0.82%
*Policy*	0	0%

^a^New strategy added during coding

The discrete strategies identified represent five of the six overarching strategy categories included in the Powell et al. [6] compilation. Quality management strategies (43%) were most common followed by planning (30%), educational (17%), restructuring (7%) and financing (2%) strategies; no strategies from the policy category were reported. The most commonly used discrete strategies were tailoring implementation plans (planning) (14%), developing quality systems (9%), using data experts (8%) and clinical supervision (8%) (all quality management strategies).

**Table 2:** Strategy use and dosage by category

	Participants involved		Person-hours	
	Median (SD)	Range	Hours	%
Planning	2.5 (1.8)	1–12	671.7	30.9
Education	2.2 (1.8)	1–12	294.7	13.5
Finance	6.2 (4.4)	1–15	55.15	2.5
Restructure	2.5 (2.5)	1–11	147.32	6.8
Quality management	2.2 (2.2)	1–13	1007.8	46.3
Policy	0	0	0	0

### Temporality

**Fig. 2:**
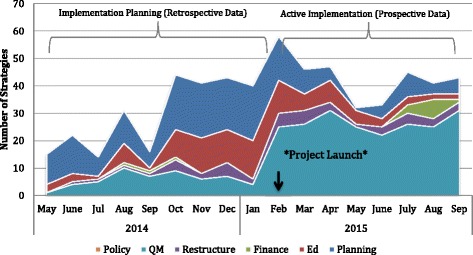
Temporality – implementation strategies used over time

Second, the number of implementation strategies used monthly was used to explore the temporality of implementation strategies (Fig. 2). In the 9 months leading up to project launch in February 2015, planning strategies were most prevalent (half of all implementation activities). Educational strategies increased during the 4 months leading up to the project launch (23–35% of the implementation strategies), and quality management strategies dominated implementation after the launch (43% of implementation activities in February 2015 and 72% during September 2015).

### Dosage – participants and hours of effort

We examined the dosage of implementation efforts based on the number of individuals involved in implementation and the total amount of time each one spent on an implementation strategy as reported in the logs. On average, multiple individuals were involved in each activity (M = 2.4, SD = 2.2), which was comparable across most strategy types except for financing strategies, which involved a greater number of participants (M = 6.2, SD = 4.4) (Table 2).

Because most activities involved multiple individuals, estimating dosage based only on their duration underestimates the amount of time invested. Instead, we estimated the person-hours (represents one hour of work by one person) invested in implementation in total, and over time. When the number of individuals participating in each activity is considered, implementation activities accounted for a total of 1594 person-hours (about 199 8-hour work days) over the observation period. Quality management activities accounted for nearly half (46.3%) and planning activities accounted for 31% of the hours of effort reported (Table 2).

**Fig. 3:**
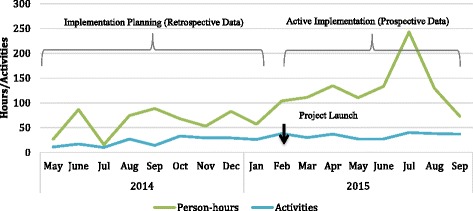
Dosage – implementation activities and person-hours over time

**Fig. 4:**
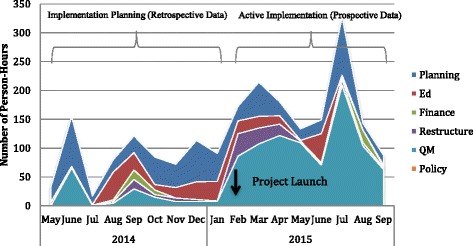
Implementation dosage by strategy category

Over time, the amount of person-hours invested in implementation increased, especially after the project launched in February 2015 (Fig. 3). A spike in planning and quality management activities over the summer (associated with discussions about the potential to scale-up the screening component to additional intake units) was accompanied by rising implementation person-hours during July and August (Fig. 4).

The leadership team reported the greatest person-hours (43.6%), followed by the assessment (28.7%) and screening implementation teams (14.4%). This may be attributed to the collaborative nature of these teams, whereby multiple individuals were involved with each implementation activity reported by leadership (M = 3.2, SD = 2.7), screening (M = 2.5, SD = 1.5) and assessment teams (M = 1.7, SD = 1.5).

### Variation in strategy use by implementation actors

**Fig. 5:**
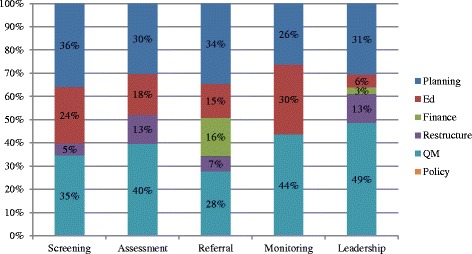
Proportion of implementation strategies used by actors

We explored variations in the types and dosage of discrete strategies by the five types of implementation actors. The highest number of strategies was reported by the screening (31.2%) and leadership teams (26.0%) (Table 3). For all teams, quality management, planning and educational strategies accounted for nearly all of the implementation strategies used (Fig. 5). All teams, except the monitoring team, also used restructuring strategies (especially restructuring records systems, structures and equipment). Notably, the referral and leadership teams were the only ones to use financing strategies (16% and 3% of activities, respectively).

**Table 3:** Implementation activities by actor

	Times used		Participants involved		Person-hours	
	f	%	M (SD)	Range	Hours	%
Screening	173	31.2%	2.5 (1.5)	1–8	211.35	14.4%
Assessment	96	17.3%	1.7 (1.5)	1–10	422.25	28.7%
Referral	61	11.0%	1.2 (0.5)	1–3	31.68	2.2%
Monitoring	80	14.4%	1.5 (0.7)	1–2	163.08	11.1%
Leadership	144	26.0%	3.2 (2.7)	1–15	640.46	43.6%

### Strategy specification

Finally, we explored how data derived from the activity logs can be used to specify the discrete implementation strategies used, the main actors, temporality and dosing, consistent with new guidelines for their specification [5]. Table 4 presents an example of specification of two discrete strategies used to implement the screening project component. Data from the activity logs was used to report the action, actor, temporality and dose, while the research team inferred strategy target, outcome and justification based on project logic models and prior literature.

**Table 4:** Example of two discrete strategies specified using Proctor et al.’s [5] guidelines (screening focused)

Strategy characteristic	How information was derived	Example 1	Example 2
Label	Activities listed in logs were coded to strategies in Powell compilation	Tailor strategies to overcome barriers (Screening)	Conduct educational meetings (screening)
Action	Activities included in logs	Meetings, emails & phone calls to plan screening work-flows, identify promising training approaches & supports	Training on use of new screening tools
Actor	Individuals associated with activities in logs	Screening implementation team Leadership team	Screening implementation team BH team leader
Target	Interpreted by the evaluation team (drawing on program logic model and plan)	Intake workers’ routines	Intake unit workers’ and supervisors’ knowledge and skills
Temporality	Timing based on dates of activities included in log	Mainly during implementation planning (Months 2–9; prior to launch) Limited use in month 10 (launch)	Prior to launch, 5 months post-launch
Dose	Based on duration, and number of individuals associated with activity on log	Total activities: 32 52.5 person-hours total	Total activities: 5 Individual dose: 1 session (2 hours) Total offered: 5 sessions (10 hours total)
Outcome	Interpreted by the evaluation team (drawing on program logic model and plan)	Feasibility	Adoption, penetration and fidelity to the screening process
Justification	Extracted from the literature based on theoretical/empirical work	Strategies must often be tailored to overcome context-specific barriers [13–16]	Training supports knowledge acquisition

## Discussion

This article describes the development, application and results of an approach to tracking implementation strategies in a multi-component project over a 17-month period. Our activity log was designed to be simple and free. Without additional compensation or incentives, our key implementation actors completed the logs consistently each month, suggesting that this approach carries minimal participant burden. Although the research team required a few hours each month for data collection and coding, our approach may still be low-cost and practical for use in implementation research and practice. We illustrate how researchers and practitioners might use this approach to generate information and specify implementation strategies. Below, we consider our method and results in the context of prior implementation research.

### Identifying discrete strategies

This strategy tracking approach identifies a broad range of discrete strategies used in research and practice, including those that occur informally or privately (e.g. building staff buy-in). In this application, we identified 45 discrete implementation strategies. Our findings reflect the broad diversity of efforts that may be used to launch complex and interdependent practice changes, consistent with other observational studies that report on real-world implementation, e.g. [44–46]. It should be mentioned that, at this stage of the project, the effectiveness of the strategies reported here is unknown – not all of these strategies might be needed or contribute equally to effective implementation. However, identifying all of the strategies used to implement an intervention is an important first step toward isolating those that are most essential. Thus, this tool might be useful in naturalistic studies or contexts where little is known yet about implementation.

Additionally, this tool highlights how often each discrete strategy is deployed, which is important for reporting and replication. In our study, planning and educational strategies were used consistently throughout implementation as ongoing activities, while restructuring and financing occurred on a limited basis. No policy strategies were used, which is consistent with other studies suggesting that implementation actors may not consider the use of these strategies or feel that changing policy and regulations in their field is feasible [42, 47]. Notably, especially after project launch, quality management strategies were used most frequently, such as creating and refining quality management systems and tools, using data experts, providing clinical supervision and sending reminders. In this study, data systems needed to be modified to gather, store and report on the information gathered from the new screens and assessments. Further, new workflows and routines were needed to facilitate secure and seamless data transfers between the child welfare agency and co-located behavioural health team. The use of quality management strategies may be most convenient or useful in implementation after planning and education have taken place and in response to barriers that emerge as frontline workers use new practices, although this warrants further testing. These findings perhaps indicate that this tool is sensitive to unique project contexts, and capable of capturing strategies that emerge in response to project contingencies.

It should also be noted that, in this study, the activities reported included meetings, informal conversations and individual work tasks, where actors used discrete implementation strategies. Thus, discrete strategies could be deployed in several different formats, generating even further variation in implementation processes, potentially influencing implementation success. These findings suggest that there may be value in drawing further distinctions between the mode of delivery (e.g. meeting) and the active ingredients (i.e. specific strategy components), mechanisms (i.e. how strategies function) and targets (i.e. what they aim to change) of implementation strategies [48].

### Temporality

Our approach also highlights the temporal ordering of implementation strategies. Like the *Stages of Implementation Completion* observational measure and other stage-based implementation process methods, our approach illustrates the progression of implementation strategies – planning strategies were used during the early periods, education featured prominently right before launch and quality management dominated during active implementation. However, this tool allows us to capture the progressive use and co-occurrence of discrete implementation strategies in a more detailed way. Thus, this approach might be appropriate to capture implementation processes, especially in studies that are grounded by stage-based implementation theories [12, 35]. Researchers and practitioners could use this information to plan ahead for when specific departments or personnel (e.g. evaluation and IT) might be activated in implementation, although additional research that makes explicit connections between strategies and stages of implementation is necessary to provide clearer guidance to stakeholders.

### Actors

We also identified variations in the strategies used by different implementation actors. In this study, all implementation groups used planning, educational and quality management strategies. However, only the leadership and referral teams used financial strategies (to implement changes to referrals and services). Thus, even within the same project or intervention, different teams and project components could require different types of implementation strategies, supporting the need to tailor strategies to address implementation determinants [15]. This particular approach might be especially useful for identifying strategies in contexts where multiple teams are working collaboratively to implement a multi-component intervention.

In addition, our approach identified actors most central to implementation. For instance, in this study, the leadership team invested a substantial amount of time and effort in implementation, suggesting their importance. These results might suggest healthy implementation conditions since strong leadership is essential for promoting positive attitudes toward new practices and creating a supportive climate for implementation [49, 50].

### Dose

This approach also allowed us to estimate the ‘dose’ of implementation strategies in terms of person-hours. Although the dosage is an estimate, our approach demonstrates how much time organisations invest in implementation. In addition to specifying the dose of specific implementation activities in reports and publications, this information can also be useful to those in the field. Agency administrators could use this information to adjust the workload for their staff, or plan ahead for future implementation efforts. Additionally, these time estimates could be useful for estimating the personnel costs associated with implementation, although we describe below several factors that may limit their precision.

### Limitations and implications for implementation strategy tracking

This article illustrates the application of a practical approach for tracking implementation strategies in practice and research that can facilitate detailed reporting. Yet, we acknowledge several limitations. To begin, self-reports of implementation activities were likely subject to reporting and recall biases, producing inaccurate estimates of the number of activities and dosage. Data on strategies used from May 2014 through January 2015 were collected retrospectively, which increased the risk for recall bias. Stakeholders often consulted their calendars for this time period but may have had difficulty recalling every implementation activity, particularly the more informal activities that are typically not documented on a calendar. Thus, dosage estimates and temporality inferences may be underestimated, especially during the earlier phases. To ensure comprehensive and consistent data collection, researchers and practitioners might begin gathering information about implementation activities from the earliest stages.

Results may also be limited by missing data. To reduce the burden of implementation and evaluation on front-line workers, we only collected implementation activities from supervisors and administrators. By excluding front-line workers, we may have missed activities such as creating reminders or making an individualised system to easily access information about the new program. Further, despite follow-up efforts, we likely missed data due to non-response and thus our data might not accurately reflect all of the implementation strategies used. It is unknown whether missing data was due to a lack of implementation activities to report or failure to respond. The activity logs requested that participants list the other stakeholders involved, perhaps reducing the extent of missing data.

Finally, there were several challenges related to using the Powell et al. [6] taxonomy to code implementation strategies. First, the Powell et al. [6] compilation did not filter implementation strategies by empirical support. Therefore, the resulting strategies captured by our approach and reported here may not be empirically supported and should not be interpreted as a template for successful implementation. Participants’ descriptions and intent of their activities were sometimes limited in detail, even when the research team followed up with participants to clarify. Second, neither our approach nor the Powell et al. [6] taxonomy captures the full extent of informal activities. For instance, activity logs may not capture informal communication among colleagues, which is known to facilitate buy-in, shared understanding and successful implementation [51–53]. These types of strategies might be more challenging to capture accurately than others that are more public (e.g. formal training) necessitating alternative tracking approaches. Additional focus groups, interviews, ethnographic or participant-observation methods conducive to uncovering these activities could complement strategy tracking efforts. Additionally, alternative strategy taxonomies (e.g. [29, 54, 55]) could be used study to code data from activity logs, which might produce different findings.

Third, objectively applying the broad strategy definitions from the compilation was challenging, requiring further discussion among the coding team to crystallise applications of each strategy within this setting. For example, ‘developing and organising quality management systems’ broadly includes efforts around planning procedures that monitor clinical practices and outcomes; in our setting, this specifically manifested as the design of an online survey platform, installation of scoring software on agency computers and an email alert system to collect, score, store and share the behavioural health screening and assessment activities. Coding the activities required project knowledge (key players, their roles and history with the program, explicit and implicit goals, barriers, timeline, etc.). However, by having coders from the evaluation team who were also involved with project planning, we introduced the potential for additional bias. Therefore, a third coder with limited knowledge of the project was brought on to build a stronger consensus around code application. To facilitate specification of strategies, especially the intended outcomes, future efforts to capture implementation strategies might include more targeted follow-up and guidance for project personnel about how they describe their activities to facilitate a more objective coding process. Likewise, coders might need training and assistance to apply strategy taxonomies [43] and ample time to build consensus around how strategies manifest in the data.

## Conclusion

This article presents a method for tracking implementation strategies based on regularly collected activity logs completed by key project personnel. In an application of this approach to an implementation project, the data were used to identify discrete implementation strategies, describe their temporal ordering, estimate dose and pinpoint critical implementation actors. This approach might be especially useful for capturing and specifying a broad range of formal and ‘behind the scenes’ implementation strategies, and promote consistent strategy reporting so that the field can better evaluate their effectiveness. These results and described limitations serve as a promising resource for guiding future assessment of implementation efforts both in clinical and research settings.

## Additional files

Additional file 1: Intervention details. (DOCX 18 kb)
Additional file 2: Activity log instructions and template. (DOCX 21 kb)
Additional file 3: Codebook and example activities. (DOCX 56 kb)
