# Supplementary material for: Tracking implementation strategies: a description of a practical approach and early findings
Source: Health Res Policy Syst. 2017 Feb 23;15:15. doi: 10.1186/s12961-017-0175-y (PMC5324332; doi:10.1186/s12961-017-0175-y)
Supplement: Additional file 1: — Intervention details. (DOCX 18 kb) [file 12961_2017_175_MOESM1_ESM.docx]

**Supplemental Materials 1**

**Intervention Details**

This study examined strategies used to implement four project components:

1. *Screening* – To improve identification of children with potential behavioral health concerns, the agency selected and implemented several developmentally tailored screening tools within intake units. Screens are conducted by child welfare workers with families whose children are entering agency custody. For purposes of this project, four intake units were selected to pilot use of these screening tools. All completed screens were routed to a co-located behavioral health assessment team for scoring; children who screen positive were subsequently administered a full clinical assessment.
2. *Assessment* – A behavioral health assessment team staffed by a local nonprofit behavioral health organization is co-located within the child welfare agency. As part of this project, new empirically validated assessment tools were implemented within the team. The assessment team conducted these assessments for children who have scored positive on the earlier screening within 10 days, and every 90 days thereafter. The assessment was intended to expand on the screening tool and provides more specific information about the child’s behavioral health service needs, inform the development of treatment recommendations, and guide the referral to treatment.
3. *Referral/Services* – Instead of diffusing referral responsibilities across a large number of case workers, this project centralized service linkage tasks. Using the assessment results, the assessment team in conjunction with a specialized referral team at the child welfare agency made a referral to local providers, and summarizes treatment and referral recommendations. These recommendations were shared with the on-going child welfare case worker, and integrated into the case plan (which is approved by the courts).
4. *Ongoing Case Monitoring* – As a result of the new screening and assessment procedures, ongoing case workers (who are responsible for servicing each case post-intake) were required to integrate the treatment recommendations into each case plan, monitor service linkages and achievement of case goals, and adjust the plan and services as needed over.

Summary of Project Component Goals, Innovations, Targets, and Actors

| Component | Purpose | Innovation/Practice Change | Target | Implementation Actors |
| --- | --- | --- | --- | --- |
| Screening | Improve identification of children and youth involved in the child welfare system with behavioral health service needs. | Behavioral health screening tools to be used for all children who enter custody:   - Childhood Trust Events Survey (CTES) - Devereux Early Childhood Assessment (DECA) - Strengths and Difficulties Questionnaire (SDQ)   Portable tablets for electronic administration.  Workflow processes for transferring completed screens to the behavioral health team. | Child welfare workers – Intake units | Leadership Team  Intake Group - Formal Group of mid-level intake administrators, and unit supervisors from the child welfare agency |
| Assessment | Provide a thorough assessment of a child’s presenting problems, strengths, and behavioral health treatment recommendations | Introduction of standardized assessment tools (and scoring software) to be administered with children who screen positive; repeated assessments every 90 days.   - Child Behavior Checklist (CBCL) - Parenting Stress Index (PSI) - Trauma Symptom Checklist for Children/Young Children (TSCC, TSCYC) | Co-located behavioral health clinicians. | Leadership Team  Assessment Group - Lead administrator, and two supervisors from the behavioral health team, |
| Referral/ Services | Target referrals to community based treatments that match children’s identified needs. | Use of new assessment findings to inform the treatment referral.  Transfer of referral responsibilities from ongoing case worker.  Formalize relationships between the child welfare agency and community-based providers | Co-located behavioral health clinicians  Specialized referral team within the child welfare agency. | Leadership Team  Services Team – informal team of 1 lead administrator, and 2 supervisors overseeing contracted services at the child welfare agency |
| Ongoing Case Monitoring | Ensure each child is connected to recommended treatment and make service adjustments as needed. | Integrate the assessment-informed treatment recommendations into the case plan.  Monitor changes in the child’s behavioral health using assessment scores. | Child welfare workers – ongoing case managers | Leadership Team  Case Monitoring Team – informal team of 2 mid-level administrators who oversee ongoing. |
