# Supplementary material for: Tracking implementation strategies: a description of a practical approach and early findings
Source: Health Res Policy Syst. 2017 Feb 23;15:15. doi: 10.1186/s12961-017-0175-y (PMC5324332; doi:10.1186/s12961-017-0175-y)
Supplement: Additional file 2: — Activity log instructions and template. (DOCX 21 kb) [file 12961_2017_175_MOESM2_ESM.docx]

**Supplemental Materials 2**

**Activity Log Instructions and Template**

This spreadsheet is designed to gather information about your Gateway CALL-related activities each month.  At the end of each month, please use the attached spreadsheet to list and describe all Gateway CALL-related activities that you participated in.  The completed spreadsheet should be sent to Hillary Robertson at [robertson.227@osu.edu](mailto:robertson.227@osu.edu) on the last working day of each month.

**Event/Activity** – Include any activities, events, efforts, or methods to change screening, assessment, referral, contracting or other type of practice for the Gateway CALL project. Examples might include (but are not limited to):

- Planning a workflow for trauma screening.
- Assessing barriers to implementing Gateway CALL.
- Training workers/assessors to use the new screening/assessment tools.
- Meeting with key personnel within FCCS who will champion the project.
- Providing consultation to workers or supervisors to improve their use of screening/assessment/referrals.
- Informing and influencing key external stakeholders to spread the word about Gateway CALL
- Revising the roles of workers, clinicians, or providers to include elements of the Gateway CALL project.
- Adjusting IT system or other data systems to assess implementation and impact of Gateway CALL.
- Development and use of a reminder system to prompt workers or clinicians to use screening or assessment tools.
- Capturing and sharing information about how Gateway CALL implementation is going.
- An email sent to workers reminding them of the procedures for screening.

***If you are unsure whether an activity should be included, include it anyway and provide us with a good description so we can best classify your efforts.*

**Date/Time** – Enter approximate dates and amount of time you spent on each activity. Estimates are fine.

**Purpose** – describe the intention of your work. This might seem redundant with the type of activity, which is ok. This is the place to describe the status or outcome of your efforts, and how they contribute to the implementation of Gateway CALL.

**Attendees** – if you participated in the event/activity with others, please note your collaborators here.

The evaluation team plans to use this information to describe the nature and amount of efforts needed to launch this type of project in a public child welfare agency, which is a requirement of the federal grant. If you feel that I need to share this spreadsheet with additional people, please let me know.

We appreciate your willingness to help us track activities! If you have any questions please feel free to contact Alicia at [bunger.5@osu.edu](https://email.osu.edu/owa/redir.aspx?SURL=vKXd-fHTX79Pg6wlsYD2s2zIGkd4aVfkrGrBSMz45CSZy3D2PZXTCG0AYQBpAGwAdABvADoAYgB1AG4AZwBlAHIALgA1AEAAbwBzAHUALgBlAGQAdQA.&URL=mailto%3abunger.5%40osu.edu).

| **Implementation Activity Log** | | | | |
| --- | --- | --- | --- | --- |
|  |  |  |  |  |
| **Event/Activity** | **Date** | **Time** | **Purpose** | **Attendees** |
| *Example: Training* | *1/1/2015* | *3pm – 5pm* | *Train Intake workers on new screening process and gain support for project* | *Intake workers and project director (*in addition to job title, please include attendees first & last names*)* |
|  |  |  |  |  |
|  |  |  |  |  |
|  |  |  |  |  |
|  |  |  |  |  |
|  |  |  |  |  |
|  |  |  |  |  |
|  |  |  |  |  |
|  |  |  |  |  |
|  |  |  |  |  |
|  |  |  |  |  |
