# Supplementary material for: Tracking implementation strategies: a description of a practical approach and early findings
Source: Health Res Policy Syst. 2017 Feb 23;15:15. doi: 10.1186/s12961-017-0175-y (PMC5324332; doi:10.1186/s12961-017-0175-y)
Supplement: Additional file 3: — Codebook and example activities. (DOCX 56 kb) [file 12961_2017_175_MOESM3_ESM.docx]

Supplementary File 3

Codebook and Example Activities

| **Strategy** | **Definition** | **Decisions/Rules** | **Example(s) and [Event ID code]** |
| --- | --- | --- | --- |
| **Plan Strategies** | | | |
| *Gather Information: Collecting information to inform the design and implementation of the project* | | | |
| Conduct local needs assessment | Collect and analyze data related to the need for the innovation; this assessment could be focused on the description of usual care and its distance from evidence based care, outcomes of usual care, opinions from stakeholders on the needs for an innovation, or on special considerations for delivering the innovation in the local context. | Focused on children/families in the system. Can include formal (e.g. mining case records) and information data analysis/information gathering. | “SACWIS Data Management: Research assistant continued to manage and clean data for the Phase I baseline assessment.” 20813 |
| Assess for readiness and identify barriers | Assess various aspects of an organization to determine its degree of readiness to implement, barriers that may impede implementation, and strengths that can be used in the implementation effort. The assessment may focus on agency finances, other services provided, community support, clinician attitudes and beliefs, organizational climate and culture, structure, and decision making styles. There are also specific measures created to assess readiness to change that could be helpful. The readiness assessment can be used to vet or eliminate implementation sites. | Focused on context, front-line workers, and other personnel; barriers to implementation, Includes formal (e.g. baseline focus groups) and informal methods for gathering this information. | “Meet with [partner organization] IT: Begin talking about how to change IT structure to accomplish the goals of [project]” 020612  “Intake Supervision Meeting: Discuss any barriers, strategies to engage families, how to communicate with staff about the project, what is coming up next, etc.” 021015 |
| Visit other sites | Visit sites where a similar implementation effort has been considered successful. | Can also include alternative opportunities for information sharing across sites (e.g. grantee meetings, conference calls) focused on shared learning. | “[Project] grantee mtg: Attending grantee mtg in DC” 030711 |
| *Select Strategies: Identify strategies, approach, and plans for implementation* | | | |
| Develop a formal implementation blueprint | Develop a formal implementation blueprint that integrates multiple strategies from multiple levels or domains (e.g., staffing, funding, monitoring) using multiple theories or the use of an explicit theoretical framework. Use and update this plan to guide the implementation effort over time. | Includes initial development of implementation plans, and workflow documents. | “Intake Meeting: Intake meeting at [project site]. The team discussed the workflow process for the screening and assessment.” 020701 |
| Tailor strategies to overcome barriers and honor preferences | Tailor the implementation effort to address barriers and to honor stakeholder preferences that were identified through earlier data collection. | Meetings, informal conversations, or other and work focused around matching implementation approach to the context; Includes brainstorming around barriers and strategies for overcoming them. | “Planning Meeting with [project site]: The team discussed the October Report and plans for sustainability ….” 021003  “Intake Supervision Meeting: Discuss any barriers, strategies to engage families, how to communicate with staff about the project, what is coming up next, etc.” 021021 |
| Stage implementation scale up | Phase implementation efforts by starting with small pilots or demonstration projects and gradually moving to system-wide rollout. | Includes meetings or work to discuss/plan for whether and how the project will be expanded within the agency or beyond. | “Intake Management Meeting:  Discussion re: [project] expanding at Intake …. Discussed expansion of pilot to four more units ….” 030921  “Various e-mails & discussions:  Planning e-mails and follow up discussions to arrange schedule for training, roll out to new units, and other details to ensure new units will be ready to go.” 030935 |
| Model and simulate change | Model or simulate the change that will be implemented prior to implementation. These efforts could involve computer simulations, walk-through simulation exercises, or modeling the overall impact of clinicians’ intentions to change their clinical behaviors. | NA | None |
| *Build Buy-In: Generate excitement and acceptance of the project.* | | | |
| Conduct local consensus discussions | Include providers and other stakeholders in discussions that address whether the chosen problem is important and whether the clinical innovation to address it is appropriate. | Includes conversations about the scope of behavioral health problems among children who have experienced maltreatment, and how the child welfare agency plays a role in addressing the problem. | “Intake Meeting: [Evaluation team] attended and presented preliminary findings from Phase I evaluation activities (youth service needs). ….” 020601 |
| Involve executive boards | Involve existing governing structures (e.g., boards of directors, medical staff boards of governance) in the implementation effort, including the review of data on implementation processes. | In this setting, governing structures included board of directors at the county child welfare agency and behavioral health organization, and liaisons at the state child welfare agency. | “Discussion with [partner organization] Administration:  Prep administration for [project] implementation, what this will require, what we will be doing, etc. Ready administration for doing this work” 021028  “Call to [state liaison] ” 020814  “Presentation on Trauma and the Family to [project site] Board of Directors” 406035 |
| Identify and prepare champions | Cultivate relationships with people who will champion the clinical innovation and spread the word of the need for it. This strategy includes preparing individuals for their role as champions. Champions can be internal or external to the organization. | Champions could be administrative leaders, or front-line workers/clinicians. | “Update [Clinical] Team: Give team updates and get them excited about the screening tool trainings” 020822 |
| Involve patients/consumers and family members | Engage or include patients/consumers and families in all phases of the implementation effort, including training in the clinical innovation, and advocacy related to the innovation effort. | NA | None |
| *Initiate Leadership: Training leaders and using leadership authority to implement* | | | |
| Recruit, designate, and train for leadership | Recruit, designate, and train leaders for the change effort. Change efforts require certain types of leaders, and organizations may need to recruit accordingly, rather than assuming that their current personnel can implement the change. Designated change leaders can include an executive sponsor and a day-to-day manager of the effort. | Efforts focused on those who manage teams that are involved with the project (team supervisor, or administrator). | “Update [Clinical] Team Supervisors: Meet with [Clinical] Team supervisors to discuss the project, update them on progress and discuss next steps” 020508  “Management discussion of [project] and review of procedure: Discussed project and procedure with staff so they could gain a better understanding of what was expected from them.” 30208 |
| Mandate change | Declare that the innovation will be implemented. | NA | None |
| *Develop Relationships: Focuses on establishment of external partnerships that will facilitate implementation.* | | | |
| Build a coalition | Recruit and cultivate relationships with partners in the implementation effort. Partnerships can develop around cost-sharing, shared resources, shared training, and the division of responsibilities among partners. This work may proceed naturally from local consensus discussions. | NA | None |
| Develop resource sharing agreements | Develop partnerships with organizations that have resources needed to implement the innovation. As an example, a group of providers could strike a relationship with a microbiology lab to conduct specialized lab work needed to implement an innovation efficiently. |  | “Contract Amendment: Received contract extension document for [referral agency] license agreement; had the document executed and scanned back to provider.” 40171 |
| Obtain formal commitments | Obtain written commitments from key partners that state what they will do to implement the innovation. | NA | None |
| Develop academic partnerships | Partner with a university or academic unit for the purposes of shared training and bringing research skills to an implementation project. | Any planning, negotiation, or consultation between the core leadership team and those at academic institutions; centered around soliciting partner’s expertise. | “Call with [Partner]:  Participated in a call with [Partner] to discuss the results of the organizational capacity survey and begin to discuss planning for the reconfiguration of the service array.” 021201 |
| **Educate Strategies** | | | |
| *Develop Materials: Compilation and development of materials used for implementation* | | | |
| Develop effective educational materials | Develop and format guidelines, manuals, toolkits and other supporting materials in ways that make it easier for stakeholders to learn about the innovation and for clinicians to learn how to deliver the clinical innovation. Create eye-catching, easy to use documents. Distill complex information into easier-to-learn components. Consider teaching skills modularly. Use different forms of media. Target messages for different audiences. | Could be materials used or distributed as part of formal training, or other materials (e.g. letter) developed to influence knowledge and practice related to the project. | “Develop slides and materials for screening tool training” 020820  “Draft Letter to Providers and Workflow Document: Prepare letter to providers detailing [project] and edit the provider services workflow document” 021220 |
| Develop a glossary of implementation | Develop a glossary to promote common understanding about implementation among the different stakeholders. | In person meetings and administrative work with the purpose of defining key terms or concepts. | “[Project] tracking training: Refining definitions and fixing drift” 030617 |
| *Educate: Disseminates information about the project and expected practice changes* | | | |
| Distribute educational materials | Distribute educational materials (including guidelines, manuals and toolkits) in person, by mail, and/or electronically. | Materials could include those formally developed for the project, or informally developed guidance (e.g. by a supervisor). | “Email to each team who was assigned a [project] case with the procedure attached:  I am sending an email to each team … who is assigned a [project] case, I let them know what to expect, ask them to again review the procedure and let them know I will be checking back in to see how things go. I also ask them to email me any feedback at any times” 30217 |
| Conduct educational meetings | Hold meetings targeted toward providers, administrators, other organizational stakeholders, and community, patient/consumer, and family stakeholders to teach them about the clinical innovation. | Includes initial formal trainings, or informational meetings for staff. Does not include one-on-one consultation sessions or supervision. | “Provider Meeting: …small group meetings that provided an overview of [project], outlined the process for communicating assessment reports with providers, and showed them a sample of what that process will look like for providers. It also helped to gain buy-in from providers and answer any questions they might have.” 021105  “Ongoing Meeting: …. These were informational presentations that provided an overview of [project] and outlined the screening and assessment process for the Ongoing Department. It was also helpful in gaining buy-in with the staff at [project site].” 21212 |
| Conduct ongoing training | Plan for and conduct training in the clinical innovation in an ongoing way. This can include follow-up training, advanced training, booster training, purposefully spaced training, training to competence, integration of off-the-job and on-the-job training, the introduction of concepts in a specific sequence to ensure mastery, and trainings based on the level of clinician knowledge. Trainings can be in-person, on the web, or technology-assisted. | Includes formal refresher trainings. Does not include one-on-one consultation sessions or supervision. | “Refresher Trauma Screening Training: Attended and administered the baseline worker survey to workers who had not yet been trained on the screening procedures.” 30125 |
| Make training dynamic | Vary the information delivery methods to cater to different learning styles and work contexts, and shape the training in the innovation to be interactive. This includes efforts to divide material into small time intervals and the use of small group breakouts, audience response systems, and other measures. | NA | None |
| Conduct educational outreach visits | Use a trained person who meets with providers in their practice settings to educate providers about the clinical innovation with the intent of changing the provider’s practice. The term academic detailing is often used synonymously. | NA | None |
| Use train-the-trainer strategies | Train designated clinicians or organizations to train others in the clinical innovation. Determine whether clinicians trained as trainers are eligible to train others as train the trainers. | NA | None |
| Provide ongoing consultation | Provide clinicians with continued consultation with an expert in the clinical innovation. This could include in-person or distance consultation and feedback on taped clinical encounters. This consultation is tailored to the clinician’s actual practice, to differentiate it from ongoing training. This feedback may be from a consultant external to the organization, which distinguishes it from clinical supervision. | May take the form of coaching; often individualized and delivered by the behavioral health assessment team supervisor. | “Follow up training on assessment tools and scoring” (between clinical coordinator and clinicians) 021030 |
| *Educate Through Peers: Strategies that target the social relationships among workers, supervisors, and project staff.* | | | |
| Inform local opinion leaders | Inform providers identified by colleagues as opinion leaders or “educationally influential” about the clinical innovation in the hopes that they will influence colleagues to adopt it. | Supervisors play a key role in influencing their workers to adopt project components; includes efforts to keep supervisors updated and informed. | “Supervision Meeting: discuss [project] briefly, let [supervisor] know that we received phase II approval” 020510  “Ongoing Management Meeting – East: Discuss [project] with Ongoing and let them know that the project "was coming." |
| Create a learning collaborative | Develop and use groups of providers or provider organizations that will implement the clinical innovation and develop ways to learn from one another to foster better implementation. This is called several things in the literature including peer consultation networks, online communities of practice, quality circles, and learning collaboratives. | NA | None |
| Shadow other clinicians | Have clinicians shadow other clinicians who are expert or knowledgeable in the clinical innovation and have implemented it. | NA | None |
| *Inform and Influence Stakeholders: Education and information targeted toward a wider stakeholder group (outside of the agency)* | | | |
| Use mass media | Use media to reach large numbers of people to spread the word about the clinical innovation. | NA | None |
| Prepare patients/consumers to be active participants | Prepare patients/consumers to be active in their care, to ask questions, and specifically to inquire about care guidelines, the evidence behind clinical decisions, or about available evidence-supported treatments. | NA | None |
| Increase demand | Attempt to influence the market for the clinical innovation to increase competition intensity and to increase the maturity of the market for the clinical innovation. | NA | None |
| Work with educational institutions | Encourage educational institutions to train clinicians in the innovation. | Use of academic partners to disseminate information about the project to other clinicians and researchers to generate interest and demand. | “Present: [Members of evaluation team] gave an oral presentation at [conference] in Tampa, FL.” 021007 |
| **Finance Strategies** | | | |
| *Modify Incentives* | | | |
| Alter incentive/ allowance structures | Work to incent the adoption and implementation of the clinical innovation. The incentive could be in the form of an increased rate of pay to cover the incremental costs associated with implementing the clinical innovation. The incentive could be through loan reduction/forgiveness to clinicians as an incentive to learn an innovation. This category of financial strategies also includes the elimination of any perverse incentives (incentives that become a barrier to receiving appropriate care). An incentive suggests the payment is tied to performing the clinical action. An allowance suggests that the clinician is not required to perform the clinical action. | NA | None |
| Use capitated payments | Pay providers a set amount per patient/consumer for delivering clinical care. This is an implementation strategy to the degree that it frees the clinician to provide services that they may have been disincented to provide under a fee-for-service structure. This may be helpful to motivate clinicians to use certain clinical innovations. | NA | None |
| Penalize | Penalize providers financially for failure to implement or use the clinical innovation. | NA | None |
| Use other payment schemes | Introduce such payment approaches (in a catch-all category) as pre-payment and prospective payment for service, provider salaried service, the alignment of payment rates with the attainment of patient/consumer outcomes, and the removal or alteration of billing limits (such as numbers of encounters that are reimbursable). These are implementation strategies to the degree that they free the clinician to provide the clinical innovation. Others motivate the clinician to provide better service. | NA | None |
| Reduce or increase patient/consumer fees | Create fee structures where patients/consumers pay less for preferred treatments (the clinical innovation) and more for less preferred treatments. | NA | None |
| *Facilitate Financial Support* | | | |
| Place on fee for service lists/ formularies | Work to place the clinical innovation on lists of actions for which providers can be reimbursed (e.g., a drug is placed on a formulary, a procedure is now reimbursable). | NA | None |
| Fund and contract for the clinical innovation | [Governments and other payers of services] issue requests for proposals to deliver the innovation, use contracting processes to motivate providers to deliver the clinical innovation, and develop new funding formulas that make it more likely that providers will deliver the innovation. | Includes the time and effort to generate the request for proposal, review applications, announce awards, and identify contracts in need of renewal/management. | “[Project] Reassessment RFP Meeting:  Discussion of the RFP for the reassessments” 020816  “[Project] Contract Discussions: Discussed current contracts and upcoming renewals with Contracts Department” 030713 |
| Access new funding | Access new or existing money to facilitate the implementation. This could involve new uses of existing money; accessing block grants; shifting funding from one program to another; cost sharing; passing new taxes; raising private funds; or applying for grants. | We used this code to denote meetings, conversations, or efforts to budget or manage funds received for this project (since these funds were new to the child welfare agency) | “Check In with [Project Officer]: [Evaluation team] attended meeting at the [project site] office. The April SAPR, Cost Study, and Funding Continuation were discussed. |
| Make billing easier | Make it easier to bill for the clinical innovation. This might involve requiring less documentation; “block” funding for delivering the innovation; and creating new billing codes for the innovation. | NA | None |
| **Restructure Strategies** | | | |
| Revise professional roles | Shift and revise roles among professionals who provide care and redesign job characteristics. This includes the expansion of roles in order to cover provision of the clinical innovation and the elimination of service barriers to care, including personnel policies. | Includes assignment of project related tasks (e.g. scoring, uploading reports in case files, case follow up). | “Conference: Discuss staff roles in monitoring the grid & workflow process” 30203 |
| Create new clinical teams | Change who serves on the clinical team, adding different disciplines and different skills to make it more likely that the clinical innovation is delivered or more successful. | NA | None |
| Change service sites | Change the location of clinical service sites to increase access; includes co-locating different services in order to better implement complex clinical innovations that require multiple disciplines or services. | NA | None |
| Change physical structure and equipment | Change the physical structure and equipment (changing the layout of a room, adding equipment). | Includes the use of new mobile technology. | “IT Meeting: These meetings were all planning meetings designed to outline the electronic process for [project site] (from feasibility of an electronic solution to [mobile device] purchase and implementation training).” 021107 |
| Facilitate relay of clinical data to providers | Collect new clinical information from the patient/consumer and relay it to the provider outside of the traditional clinical encounter to prompt the provider to use the clinical innovation. Examples might include depression scores from an instrument administered in the waiting room or telephone transmission of blood pressure measurements. | NA | None |
| Change records systems | Change records systems to allow better assessment of implementation or of outcomes of the implementation. | Can include planning activities, as well as actual modifications to existing the records system. (may overlap with Develop and organize quality monitoring systems) | “Work on Transfer Log Checklist: Add new section to Transfer Log. Work with Data Management to add this to the transfer log.” 021032  “Meet with [partner organization] IT: Discuss data sharing issues and integration of systems” 021228 |
| Start a purveyor organization | Start a separate organization that is responsible for disseminating the clinical innovation. It could be a for-profit or non-profit organization. It could be “licensed” by a university if the innovation was born within an academic setting. | NA | None |
| **Quality Management Strategies** | | | |
| Develop and organize quality monitoring systems | Develop and organize systems and procedures that monitor clinical processes and/or outcomes for the purpose of quality assurance and improvement. This includes developing systems for monitoring through peer reviews, collecting data from patients/consumers, clinicians, and supervisors, and using administrative and electronic record data. This category of strategies also includes the design of disease-specific clinical registries, where clinical information and tools (graphical representations, real-time report cards, comparisons to benchmarks, etc) are available to care team members. These systems may inform audit and feedback strategies. | Focuses on infrastructure – development or refinement of data systems. Includes the development of new systems (e.g. tracking log), as well as modifications to existing systems (may overlap with Change Records Systems” | “[Partner organization] IT meetings: Working on ways to capture data in our EMR and to improve workflow and automate scoring/reporting” 30246  “[Project] tracking Log: Repeated issues with tracking log disappearing and not saving information; took opportunity to enhance data collected in columns” 30304  “Created Excel spreadsheet to track [project] cases assigned to West Region: Document enables the Associate Directors to count and monitor the [project] cases assigned to West Region.” 30414 |
| Develop tools for quality monitoring | Develop, test, and introduce into quality-monitoring systems the right input – the appropriate language, protocols, algorithms, standards, and measures (of processes, patient/consumer outcomes, and implementation outcomes) that are often specific to the innovation being implemented. | Focuses on data - entry, cleaning, management, analysis (whereas Develop and Organize Systems focuses on infrastructure). Includes the development of SQL language for data extraction, and reviewing data quality. | “SACWIS Data Management: Research Assistant continued to manage and clean SACWIS data for the Phase I baseline assessment.” 020813  “IT solutions: …reports for incomplete screens with [project site] IT; … improve documentation tools in [partner organization] EMR” 30433 |
| Audit and provide feedback | Collect and summarize clinical performance data over a specified time period and give it to clinicians and administrators in the hopes of changing provider behavior. The summary may include recommendations. The information may have been obtained from a variety of sources, including medical records, computerized databases, observation, or feedback from patients. A performance evaluation could also be considered as audit and feedback if it included specific information on clinical performance. | Can take on different forms; includes review of performance (are children screened who should be screened?), as well as data quality (are data entered accurately?). Information may be relayed back to supervisors as well as leadership team. | “Finalize April Quarterly Report: [Evaluation team] finalized the April Quarterly report and submitted it to [project site].” 30446  “Data Analysis: Ran SQL and researched Access DB for details related to screens completed per request of [project] consultant” 030607 |
| Remind clinicians | Develop reminder systems designed to prompt clinicians to recall information or use the clinical innovation. The reminder could be patient or encounter specific, provided verbally, on paper, or on a computer screen. Computer-aided decision support and drug dosages are included in this strategy. | Can also include reminders to non-clinical staff and supervisors who are implementing the project. | “Problem solving through phone contact and e-mails:  …. Reminder to pilot unit supervisors to not process [cases assigned to managed care provider] as project cases. ….” 30224  “Emails to staff who received [project] cases: Ensuring staff have information on [project] and a reminder on how these cases are different” 30324 |
| Use advisory boards & workgroups | Involve multiple kinds of stakeholders in a group to oversee implementation efforts and make recommendations. | Includes seeking advice from other stakeholders such as the program officer; the purpose of these activities is to solicit feedback, information, and advice for assessing and improving implementation | “Leadership Meeting:  [Evaluation team] participated in the Leadership Meeting at [project site]. The team discussed the roll-out of the new screening and assessment procedures. In addition, they discussed the results of the baseline worker survey, the implementation activity log, and the training procedures for new workers in the pilot units.” 30219 |
| Obtain and use patient/consumer and family feedback | Use mechanisms to increase patient/consumer and family feedback on the implementation effort. This could include complaint forms, or methods to funnel feedback to advisory boards. | NA | None |
| Obtain and use worker feedback** | Use mechanisms to solicit front-line worker and supervisor feedback on the implementation effort. Includes surveys, focus groups, participatory meetings, or other methods for funneling feedback to the advisory board. | None | “[Project] Intake Meeting: [Research coordinator] attended the Intake meeting. The team discussed the screen and assessment process and received feedback from the Intake units, Ongoing units, and [clinical] team on barriers/facilitators.” 30443  “Conduct Focus Group” 030736 |
| Centralize technical assistance | Develop and use a system to deliver technical assistance focused on implementation issues. This could be the designation of a lead technical assistance organization (could also be responsible for training). The lead technical assistance entity can develop other mechanisms (e.g., call-in lines or web sites) to share information on how to best implement the clinical innovation. | Focus is on centralizing information – compiling all information and project assistance in one place. Can vary in terms of the technical assistance form (ranging from a person or persons to written information). | “Discussion with IT about services locator site: To gain knowledge about things to consider for the services locator site that will house all the information on [project]” 30202 |
| Provide clinical supervision | Provide clinicians with ongoing supervision. Provide training for clinical supervisors who will supervise clinicians who provide the innovation. | Used to identify meetings between front line workers and their supervisors to discuss project issues. May include supervision around project-related clinical and administrative issues. | “Supervision/Training: Review of cases and integration of [project] assessment tools into written narrative” 30322  “Consultation with caseworker:  Discussed circumstances of child’s involvement with [project site] and need to conduct a [project] screen” 030732 |
| Intervene with patients/consumers to enhance uptake and adherence | Intervene with patients/consumers to increase uptake of and adherence to clinical treatments. This includes consumer/patient reminders and financial incentives to attend appointments. | NA | None |
| Purposefully re-examine the implementation | Obtain commitment from stakeholders to use monitoring to adjust practice and strategies to continuously improve the implementation effort and delivery of the clinical innovation. | Deliberate discussion of implementation challenges and successes; use this information to adjust implementation plans as needed. Usually takes the form of meetings. | “Weekly review of barriers and success: Developing new strategies to enhance [project] with team” 030926  “[Project] Leadership Meeting: Leadership meeting (discussing project issues more broadly), and a key leadership meeting (discussing [new clinical team] transition more specifically)” 40131 |
| Conduct cyclical small tests of change | Implement changes in a cyclical fashion using small tests of change before taking changes system-wide. Results of the tests of change are studied for insights on how to do better. This process continues serially over time and refinement is added with each cycle. Two common small tests of change cycling strategies are “Plan-Do-Study-Act” (PDSA) from Deming’s quality management work and six sigma’s Define- Measure-Analyze-Improve-Control (DCMA) sequence. | NA | None |
| Use data warehousing techniques | Integrate clinical records across facilities and organizations in order to facilitate implementation across systems. | Planning and work needed to integrate child welfare (SACWIS) and behavioral health/Medicaid records; includes efforts related to data protection and security. | “Call with [college and university] IT: [Research coordinator] and IT staff discuss future data sharing and storage of protected health information.” 020812 |
| Use an improvement/ implementation advisor | Seek guidance from experts in implementation. This could include consultation with outside experts such as university-affiliated faculty members, or hiring quality improvement experts or implementation professionals. | NA | None |
| Use data experts | Involve, hire and/or consult experts in data management to shape use of the considerable data that implementation efforts can generate. | Can include internal (within the child welfare agency) and external (e.g. academic partner) data personnel. | “Conference Call [: [Evaluation team] participated in a conference call with [collaborator on campus] to discuss the [project] research dataset (merged SACWIS and Medicaid Records).” 021101  “Data Interrogation: Reviewed data needs and assisted with establishment of SharePoint data housing for [project] screening and assessment tools, and established SharePoint views to see/check the data; shared information with clinician” 30329 |
| Capture and share local knowledge | Capture local knowledge from implementation sites on how implementers and clinicians made something work in their setting and then share it with other sites (see centralized technical assistance and learning collaboratives). | Focus on external dissemination (outside of the child welfare agency). | “Phone call: Discussion with another county on [project]” 30439  “Metro Meeting: Convening of [other public child welfare agencies] across the state to discuss updates, Updated on [project]” 31148 |
| Organize clinician implementation team meetings | Develop and support teams of clinicians who are implementing the innovation and give them protected time to reflect on the implementation effort, share lessons learned, and support one another’s learning. | Formal or informal meetings among front-line child welfare workers or behavioral health clinicians. | “Unit Meeting: Discuss [project] feedback with the group in regards to any challenges, who has completed an assessment yet, concerns with the surface or the connection while out in the field.”  31159 |
| Plan for outcome evaluation ** | Efforts related to collaborative design and preparation for the evaluation of intervention outcomes; work with partners to inform and tailor evaluation methods, indicators, and procedures to project context. | Applies to meetings, calls, and work. | “Evaluation team participated in a call with [organization] IRB and OSU IRB to determine the next steps for the outcomes study protocol… it was determined that the team needed to submit a short form application to [organization] IRB.” 304430  “Meet with [NAME] and {NAME]” from [organization] evaluation department to discuss SACWIS data and review projections” 30836 |
| **Policy Context** | | | |
| Change accreditation or membership requirements | Strive to alter accreditation standards so that they require or encourage use of the clinical innovation. Work to alter membership organization requirements so that those who want to affiliate with the organization are encouraged or required to use the clinical innovation. | NA | None |
| Change liability laws | Participate in liability reform efforts that make clinicians more willing to deliver the clinical innovation. | NA | None |
| Create or change credentialing and/or licensure standards | Create an organization that certifies clinicians in the innovation or encourages an existing organization to do so. Change governmental professional certification or licensure requirements to include delivering the innovation. Work to alter continuing education requirements to shape professional practice toward the innovation. | NA | None |

**New strategy added during coding
